# A New Era for Calcium Pyrophosphate Deposition Disease Research: The First-Ever Calcium Pyrophosphate Deposition Disease Classification Criteria and Considerations for Measuring Outcomes in Calcium Pyrophosphate Deposition Disease

**DOI:** 10.3390/gucdd2010005

**Published:** 2024-02-05

**Authors:** Sara K. Tedeschi

**Affiliations:** Division of Rheumatology, Inflammation, and Immunity, Department of Medicine, Brigham and Women’s Hospital, Boston, MA 02115, USA

**Keywords:** CPPD, calcium pyrophosphate, classification criteria, outcomes measurement

## Abstract

Calcium pyrophosphate deposition (CPPD) disease is a crystalline arthritis that was described more than 60 years ago, yet our knowledge about this condition greatly lags behind other forms of arthritis. This is an exciting era for CPPD disease as a robust framework for CPPD clinical research has been established. The American College of Rheumatology (ACR) and EULAR co-sponsored the development of the first-ever classification criteria for CPPD. The Outcomes Measures in Rheumatology (OMERACT) CPPD Ultrasound Subtask Force developed and validated definitions for ultrasonographic findings of CPPD, and the OMERACT CPPD Working Group is establishing a core outcome domain set for this crystalline arthritis. This review focuses on key elements of the 2023 ACR/EULAR CPPD disease classification criteria and considerations for measuring outcomes in CPPD disease.

## 1. Introduction

Calcium pyrophosphate deposition (CPPD) disease is a crystalline arthritis that was described more than 60 years ago, yet our knowledge about this condition greatly lags behind other forms of arthritis. Targeted treatments to prevent or remove calcium pyrophosphate (CPP) crystals from the joint do not exist, which is likely both a cause and consequence of the general lack of attention to CPPD over the past decades. The most common clinical manifestations of CPPD include acute mono/oligoarthritis, osteoarthritis with radiographic chondrocalcinosis (sometimes in joints that are atypical for primary osteoarthritis), and chronic inflammatory arthritis attributed to CPP crystals. Uncommon (or perhaps under-recognized) manifestations include crowned dens syndrome, tophaceous CPP deposits at the temporomandibular joint, and involvement of the spinous ligaments.

Ryan and McCarty proposed diagnostic criteria for CPPD in the 1960s [1]. In this framework, a definite diagnosis of CPPD required both the presence of synovial fluid CPP crystals visualized by polarized light microscopy and radiographic evidence of chondrocalcinosis. The sensitivity and specificity of these diagnostic criteria were not validated and it remains unclear what portion of patients with CPPD are accurately identified using this framework. Importantly, diagnostic criteria are intended to aid in clinical diagnosis and not to identify patients for inclusion in research studies.

This is an exciting era for CPPD disease as a robust framework for CPPD clinical research has been established. The American College of Rheumatology (ACR) and EULAR co-sponsored the development of the first-ever classification criteria for CPPD. The Outcomes Measures in Rheumatology (OMERACT) CPPD Ultrasound Subtask Force developed and validated definitions for ultrasonographic findings in CPPD, and the OMERACT CPPD Working Group is establishing a core outcome domain set for this crystalline arthritis. These developments provide a clear vision for patients to be included in trials and observational studies of CPPD, and what should be measured in such studies. Ultimately, this research framework will inform our understanding of the natural history and long-term consequences of CPPD disease and optimal treatment strategies.

This review focuses on key elements of the 2023 ACR/EULAR CPPD disease classification criteria and considerations for measuring outcomes in CPPD disease.

## 2. What Types of Patients Are Classified as Having CPPD Disease by the 2023 ACR/EULAR CPPD Disease Classification Criteria?

Classification criteria are intended to capture the vast majority of individuals with a given condition (i.e., they are highly sensitive) while also avoiding the classification of individuals with mimicking conditions (i.e., they are highly specific). Patients fulfilling classification criteria generally have common features of a particular disease, which facilitates comparisons across studies. Though the classification criteria are not intended to aid in clinical diagnosis, they highlight some of the constructs that experts in CPPD disease consider most relevant. Clinicians can thus look to the CPPD disease classification criteria for elements which, if present in a patient, could point toward a clinical diagnosis of CPPD disease such as chondrocalcinosis, positive synovial fluid aspirate, and episodes of acute monoarthritis in the knee or wrist. However, it is not appropriate to use the scoring system to diagnose a patient with CPPD disease.

The 2023 ACR/EULAR CPPD disease classification criteria are intended to classify patients with symptomatic CPPD disease, meaning joint pain, swelling, or tenderness in a peripheral joint or at an axial joint in the case of crowned dens syndrome [2]. If another medical condition fully explains these symptoms, then the patient should not be considered for classification as CPPD disease. However, determining whether another condition fully explains the symptoms can be difficult as CPPD frequently co-occurs with osteoarthritis, and sometimes with rheumatoid arthritis, gout, and other rheumatic diseases. The investigator applying the criteria must exercise personal judgment when deciding whether to proceed with classification. For example, in a patient with knee pain, chondrocalcinosis of the knee, and end-stage radiographic osteoarthritis, it may be difficult to determine whether the knee pain is in part due to CPPD disease or if osteoarthritis explains all of the symptoms. If at least some symptoms are thought to be due to CPPD disease then one should proceed with the classification process to see if the patient fulfills the criteria. There is an element of subjectivity at play, as the investigator applying the classification criteria must exercise judgment as to whether all symptoms are attributable to another condition. Biomarkers (not yet identified) that distinguish between symptoms from CPPD disease and symptoms from other forms of arthritis would be quite useful to aid in applying this exclusion criterion.

### 2.1. Individuals with Calcium Pyrophosphate Crystals in Synovial Fluid

Individuals with CPP crystals in synovial fluid or on tissue histopathology are classified as CPPD disease as long as the aforementioned elements are met. Synovial fluid (or tissue) CPP crystals were considered to be entirely specific for CPPD disease, and thus are sufficient for classification. This assumes that the synovial fluid or tissue specimen was obtained from a symptomatic joint that was not completely explained by another condition. A degree of individual judgment is exercised here, and future studies testing the classification criteria’s sensitivity and specificity in different clinical settings will be important. A patient with both gram-positive cocci and CPP crystals in the same synovial fluid sample may or may not be classified as CPPD disease, for example, depending on the investigator’s opinion as to whether septic arthritis fully explains all of the joint symptoms. Most clinicians would attribute joint pain and swelling to septic arthritis, but if the patient had pre-existing osteoarthritis and chondrocalcinosis of the affected joint then uncertainty about symptom attribution may exist. A patient with end-stage osteoarthritis undergoing joint replacement with CPP crystals identified on histopathology may or may not be classified, depending on the investigator’s judgment as to whether osteoarthritis explains all of the joint symptoms.

### 2.2. Individuals with Crowned Dens Syndrome

In general, classification criteria for rheumatic diseases may not capture individuals with rare manifestations because certain rare features may not be perceived as important enough to be retained during the lengthy criteria development process involving investigators, senior clinicians, and methodologists. For example, the 2019 EULAR/ACR Systemic Lupus Erythematosus classification criteria would not classify someone with positive ANA, positive double-stranded DNA antibody, and lupus pneumonitis as the sole clinical manifestation; pneumonitis represents a rare feature in SLE and was not included in the classification framework.

Crowned dens syndrome is an exception to this premise, as individuals with crowned dens syndrome are classified as CPPD disease as long as another explanation for the clinical symptoms (e.g., meningitis or polymyalgia rheumatica) and imaging findings are not thought to be more likely. Although crowned dens syndrome is a relatively rare manifestation of CPPD disease, experts involved in developing the criteria considered the combination of clinical and imaging findings to be quite specific for CPPD disease such that it was included as a sufficient criterion in the classification system. Many individuals with crowned dens syndrome also have other manifestations of CPPD disease, though these would not be considered nor required for classification in this instance.

### 2.3. Individuals without Joint Aspiration (or without Synovial Fluid CPP Crystals)

What about individuals with joint symptoms, where another condition does not fully explain the symptoms, and who do not have synovial fluid/tissue CPP crystals or crowned dens syndrome—how can they fulfill CPPD classification criteria? Consider an elderly patient with an episode of acute inflammatory arthritis of the wrist and chondrocalcinosis of the triangular fibrocartilage complex on X-ray; is this person classified as CPPD disease? It depends. A major consideration is the number of joints with chondrocalcinosis, which is a large driver of whether the threshold score for classification is met (Table 1). The number of peripheral joints with chondrocalcinosis is, in turn, partly dependent on the number of joints imaged. Other highly weighted features include having typical episodes of acute inflammatory arthritis, especially in the knee or wrist. Persistent inflammatory arthritis not otherwise explained also provides a fair amount of weight.

To achieve the threshold score for classification as CPPD disease, an individual generally must have inflammatory arthritis (either acute or persistent) and imaging evidence of CPPD. Depending on the number of joints with imaging evidence of CPPD and the characteristics of the inflammatory arthritis, other features may be required for classification such as age >60 years, related metabolic diseases (such as hyperparathyroidism), and osteoarthritis of particular hand or wrist joints. A convenient online calculator with a complete list of features and weights is available at https://bblinks.live/acr-classification-criteria-for-cppd-disease (accessed on 17 November 2023).

## 3. How Well do the 2023 ACR/EULAR CPPD Disease Classification Criteria Identify Individuals with CPPD Disease?

Whereas synovial fluid crystal analysis for monosodium urate crystals is a gold standard for gout, the same is not true for synovial fluid analysis in CPPD disease. Synovial fluid crystal analysis could be considered a “silver standard” for CPPD disease, as CPP crystals are notoriously challenging to visualize given their small size and weak or absent birefringence. For this reason, the absence of synovial fluid CPP crystals on one occasion received a slight negative weight rather than a large negative weight, as it is possible that synovial fluid crystal analysis could produce a false negative due to challenges with crystal identification. (By contrast, two or more synovial fluid aspirates negative for CPP crystals received a larger negative weight as the chance of two false negatives was considered less likely.) Additionally, synovial fluid CPP crystals can be observed in joints that do not have symptoms attributable to CPPD disease.

The CPPD classification criteria project included a derivation cohort and an independently assembled validation cohort, both of which included de-identified patient profiles. Decisions about whether each patient profile represented CPPD disease or not (i.e., controls) were reached using the clinical assessment of the physician that submitted the profile and, when uncertainty existed, adjudication by two independent experts.

The 2023 ACR/EULAR CPPD classification criteria framework demonstrated excellent sensitivity of 99.2% and specificity of 92.5% in the validation cohort. When considering these results, it is worth noting that only those patient profiles that were clearly “definite CPPD disease” or “definitely not CPPD disease” (controls) were included in the validation study. A large portion of patient profiles submitted to the validation cohort could not be adjudicated one way or the other with the available data and were omitted from the analysis. Future studies assessing the sensitivity and specificity of the CPPD disease classification criteria must carefully consider the method for determining CPPD disease status. For example, studies evaluating the sensitivity and specificity among patients with additional rheumatologic diagnoses—in which case attribution of symptoms can be particularly challenging—would be of interest.

## 4. Classification and Considerations for Clinical Trial Design

As CPPD treatment trials are designed in years to come, the ACR/EULAR criteria will likely play a prominent role as entry criteria. However, individual studies may elect to focus on particular phenotypes or clinical manifestations of CPPD disease. For example, a trial focused on reducing joint swelling may exclude patients classified as CPPD due to crowned dens syndrome if the individual would not have otherwise fulfilled the classification criteria, because joint swelling is not a relevant outcome for crowned dens syndrome alone. Alternately, trials could require a minimum set of clinical criteria to be fulfilled at enrollment, such as swelling of a peripheral joint—thereby allowing an individual classified by crowned dens syndrome to participate as long as that clinical criterion is met.

Although the classification criteria are intended to identify patients with high confidence for CPPD disease, the particular mix of clinical, laboratory, and radiographic features can differ. Clinical evidence of synovitis is one such feature. Patients with synovial fluid CPP crystals or crowned dens syndrome (i.e., sufficient criteria) may not have had synovitis (joint warmth, tenderness, and swelling) on physical examination of peripheral joints. A patient with CPP crystals aspirated from a cool, swollen knee with low synovial fluid white blood cell count (note, synovial fluid cell count is not part of the classification criteria) could be quite clinically different from a patient with persistent inflammatory arthritis and chondrocalcinosis in multiple joints. Inclusion and exclusion criteria for individual clinical studies will allow flexibility in determining which subset of patients fulfilling CPPD disease classification criteria will participate in the study.

## 5. Linking the Classification Criteria to Clinical Research Outcomes

The 2023 ACR/EULAR CPPD disease classification criteria were developed independently from the work being conducted by the OMERACT CPPD Working Group [3–6]. However, the processes share some similarities. Both began with a comprehensive literature review to identify candidate features to be considered as classification items, or outcomes reported in clinical trials [3,7]. Both groups included Patient Research Partners, although to a greater extent in the OMERACT CPPD Working Group whose scope also included qualitative interviews with patients and caregivers to understand the lived experience with CPPD disease and to generate additional potential outcome domains.

Given that many of the same studies were identified in the literature reviews, many disease constructs would be expected to overlap between classification features and outcomes to be measured in clinical studies. The presence of CPP crystals in synovial fluid and imaging evidence of CPPD are central constructs in CPPD disease, though their relevance as clinical outcomes is still being considered. An in-depth discussion on outcome measures in CPPD disease has been recently published [8].

Acute CPP crystal arthritis is the most commonly recognized manifestation of CPPD disease, and many patients with this clinical presentation will be classified as CPPD disease (depending on the particular features if joint aspiration is not performed). Treating an individual episode of acute CPP crystal arthritis could be the intent of some short-term studies while preventing episodes of acute CPP crystal arthritis could be the focus of some long-term studies. The OMERACT CPPD Working Group voted in favor of developing core outcome domains separately for long-term studies and short-term studies, recognizing that the elements to be measured in studies of individual episodes of acute CPP crystal arthritis may differ from those measured in long-term studies [4].

Potential outcome domains mirror some of the constructs relevant to classification, while other outcomes are not directly related to features for CPPD disease classification. The presence of synovial fluid CPP crystals is central to classification, while it may be only mildly important as an outcome measure in part because many studies may not require joint aspiration during follow-up. Imaging evidence of CPPD is very important for classification, though it may be only moderately important as an outcome in CPPD disease trials; currently, measures to remove CPP crystals from cartilage do not exist, though the progression of chondrocalcinosis over time may be of interest in understanding the pathophysiology of disease and its relationship with osteoarthritis. Joint pain is one of the elements necessary to be considered for classification (unless joint swelling is present) and will likely be considered a very important outcome measure.

## 6. Proposed Next Steps in Defining Outcomes in CPPD

At the time of this writing, the OMERACT CPPD Working Group is currently conducting a Delphi exercise among investigators, clinicians, patients, and other stakeholders to determine which of these and other outcome domains are most important to measure in short-term and long-term trials. Joint pain, joint inflammation, and imaging abnormalities may be among the final outcome domains, in addition to quality-of-life measures and healthcare utilization. Because validated outcome measures for CPPD disease do not currently exist, the development and validation of outcome measures will be critical before large clinical trials are conducted.

### 6.1. Clinical Outcomes

Patient-reported outcomes (PROs) capture features of disease that are key to the lived experience of disease and cannot be assessed by clinicians. Testing and/or adapting PROs used in osteoarthritis, rheumatoid arthritis, and gout—including tender and swollen joint counts, Health Assessment Questionnaire (HAQ), PROMIS pain score, and others—seems a logical next step in developing the framework for CPPD disease research.

Joint pain will likely be a primary outcome of short-term studies of individual flares of acute CPP crystal arthritis. A recently randomized clinical trial of colchicine versus prednisone for treating acute CPP crystal arthritis used the change in joint pain as the primary outcome [9]. It remains unclear what minimum pain score, or what change in pain score, is clinically relevant in CPPD disease as this metric has not yet been evaluated specifically in CPPD populations.

Defining a flare of acute CPP crystal arthritis will be important for long-term studies that include flare rates as an outcome. A provisional definition of acute CPP crystal arthritis flares has been proposed (EULAR 2023 abstract presentation), largely building off elements of a patient-reported definition for gout flares [10,11]. This provisional definition also included elements that had been identified through qualitative patient and caregiver interviews by the OMERACT CPPD Working Group as potential outcome domains to be measured in future clinical trials [6]. Future studies to validate the components of this acute CPP crystal arthritis flare definition are needed.

Not all characteristics of gout translate to CPPD disease. Flares of CPPD disease may include elements beyond just acute CPP crystal arthritis flares. For example, patients with chronic CPP crystal inflammatory arthritis—which clinically resembles rheumatoid arthritis—may experience flares that have features distinct from acute CPP crystal arthritis flares and may be more akin to a rheumatoid arthritis flare. Future work to define elements of CPPD flares may include a literature review for features of flares and validation of flare definitions in prospective clinic-based cohorts.

### 6.2. Imaging Outcomes

The OMERACT Ultrasound CPPD Subtask Force developed and validated ultrasound features characteristic of CPPD in the knee, wrist, and other joints [12,13]. Whether these ultrasound features change over time has not been investigated to this writer’s knowledge. Similarly, longitudinal studies of the progression of chondrocalcinosis are few to none. Understanding the rate at which CPPD becomes detectable or progresses on imaging would be important as a first step before testing interventions that might slow or reverse imaging evidence of CPPD.

Imaging outcomes relevant to CPPD disease might include the development of CPPD in joints without CPPD at baseline; CPPD burden (volume and location) in individual joints; and radiographic progression of osteoarthritis. Indeed, several large cohort studies have investigated whether the baseline presence of chondrocalcinosis of the knee relates to the progression of osteoarthritis on MRI imaging, with mixed results [14–16]. A recent abstract presenting the EULAR Crystalline Imaging Guidelines recommended against serial joint imaging in routine clinical care of patients with CPPD unless there is an “unexpected change in clinical characteristics” [17]. However, in research settings, serial imaging may be an informative outcome; sensitivity to change in ultrasound in particular will be of interest as a future topic of investigation.

## 7. Conclusions

After decades of standing in the shadow of gout, CPPD disease is moving into the limelight. We are at a moment when clinical trials in CPPD disease will be more comparable due to validated CPPD classification criteria with excellent performance characteristics, and a core set of features to be measured in trials is being crafted. Work that lies ahead includes developing or adapting PROs and other clinical and imaging outcome measures to ensure they are sensitive to change in CPPD and accurately represent the lived experience with the disease. Engagement with the pharmaceutical industry will be key in conducting CPPD disease clinical research; to date, interest in drug development for CPPD disease and sponsorship of clinical research has been sorely lacking. We know how to identify patients with CPPD disease and are finalizing what should be studied; the future for treating this common crystalline arthritis is bright.

## Figures and Tables

**Table 1.** Most influential (most highly weighted) features in the 2023 ACR/EULAR CPPD Disease Classification Criteria.

Sufficient Criteria *	Positively Weighted Features (Leaning Toward Classification)	Negatively Weighted Features (Leaning Away from Classification)
Synovial fluid (or tissue histopathology) positive for calcium pyrophosphate crystals	Imaging evidence of CPPD in 1 or more peripheral joints regardless of symptoms using any modality ** (additional weight if 2–3 joints, and even greater weight if 4+ joints)	Synovial fluid crystal analysis negative for CPP crystals on 2 or more occasions
Crowned dens syndrome (characterized by clinical and imaging features)	Typical episode(s) of acute inflammatory arthritis (having more than one episode receives more weight than just one episode)	
	Imaging evidence of CPPD in a symptomatic peripheral joint using any modality **	
	Knee or wrist affected by typical episode(s) of acute inflammatory arthritis	
	Persistent inflammatory arthritis without another explanation	

* Sufficient for classification as CPPD disease without considering other features, provided that joint symptoms are present (entry criterion) and another condition does not fully explain symptoms (exclusion criterion);

** Imaging modalities include conventional radiograph, ultrasound, computed tomography (CT), or dual-energy CT.

## Data Availability

No new data were generated for this manuscript.
